# Characterization of the Virome in Mosquitoes Across Distinct Habitats in the Yucatán Peninsula, Mexico

**DOI:** 10.3390/v17060758

**Published:** 2025-05-26

**Authors:** Erika N. Hernández-Villegas, Hugo G. Castelán-Sánchez, Andres Moreira-Soto, Ana Laura Vigueras-Galván, Marco A. Jiménez-Rico, Oscar Rico-Chávez, Stephany Rodríguez-González, María José Tolsá-García, David Roiz, Paola Martínez-Duque, Roger Arana-Guardía, Omar García-Súarez, Moisés Zamora Jiménez, Luisa I. Falcón, Benjamin Roche, Rosa Elena Sarmiento-Silva, Audrey Arnal, Jan Felix Drexler, Gerardo Suzán

**Affiliations:** 1 Posgrado en Ciencias de la Salud y Producción Animal, Facultad de Medicina Veterinaria y Zootecnia, Universidad Nacional Autónoma de México, Mexico City 04510, Mexico; 2 Facultad de Medicina Veterinaria y Zootecnia, Universidad Nacional Autónoma de México, Mexico City 04510, Mexico; 3 International Joint Laboratory UNAM/IRD ELDORADO, Merida 97302, Yucatan, Mexico; 4 Department of Pathology and Laboratory Medicine, Western University, London, ON N6A 3K7, Canada; 5 Institute of Virology, Charité–Universitätsmedizin Berlin, 10117 Berlin, Germany; 6 Tropical Disease Research Program, School of Veterinary Medicine, National University, Heredia 86-3000, Costa Rica; 7 Posgrado en Ciencias Biológicas, Instituto de Ecología, Universidad Nacional Autónoma de México, Mexico City 04510, Mexico; 8 MIVEGEC, IRD, CNRS, Université de Montpellier, 34090 Montpellier, France; 9 AECOM, Mexico City 01219, Mexico; 10 Laboratorio de Ecología Bacteriana, Instituto de Ecología, Unidad Mérida, Universidad Nacional Autónoma de México, Merida 04510, Yucatan, Mexico; 11 German Centre for Infection Research (DZIF), Inhoffenstraße, 38124 Braunschweig, Germany

**Keywords:** virome, mosquitoes, Mexico, metagenomic, insect-specific virus

## Abstract

Human activities and land use changes have a major impact on the distribution and diversity of mosquito vectors and their associated viruses. This study describes the diversity and differential abundance of viruses associated with mosquito species from four habitats of the Yucatan Peninsula, Mexico. Using next-generation sequencing (NGS), we analyzed 61 genomic libraries belonging to 20 mosquito species to characterize the viral community. A total of 16 viral species were identified, representing 14 different viral families. Most identified viruses were associated with insects, plants, and fungi. Additionally, vertebrate associated viral families, including *Herpesviridae*, *Peribunyaviridae*, *Nairoviridae*, and *Arenaviridae*, were detected in mosquitoes from urban habitats. Notably, insect-associated viruses like *Hubei mosquito virus 4* and *Hubei virga-like virus 2* were identified, along with the first report of *Mercadeo virus* in Mexico. Variations in viral community composition were primarily driven by mosquito species, with species of the same genus maintaining similar viromes despite occupying different habitats. These findings reinforce that intrinsic traits of mosquito species play a key role in shaping viral community composition. To our knowledge, this is the first study that describes the viral community in mosquitoes in Yucatan Peninsula, Mexico. This study provides essential baseline data for the surveillance of mosquitoes and associated viruses from a biodiverse tropical region that faces strong land use modifications.

## 1. Introduction

The emergence and re-emergence of vector-borne diseases are a worldwide public health concern. In 2020, the World Health Organization estimated that they account for more than 17% of all infectious diseases worldwide and are responsible for more than 700,000 deaths per year [1]. They overwhelm healthcare systems, especially in tropical and subtropical areas, where landscape changes influence pathogen dynamics due to human activity [2,3]. Mosquitoes are of particular concern as they are potential vectors of arboviruses (arthropod-borne viruses) such as dengue virus (DENV), chikungunya virus (CHIKV), West Nile virus (WNV), and Zika virus (ZIKV) [4,5,6,7,8]. Mosquitoes can also transmit pathogens to humans, mammals, birds, amphibians, and reptiles [9,10].

Recent studies based on next-generation sequencing (NGS) techniques have shown that mosquitoes and other insects harbor a wide variety of viruses, including both known and novel taxa [11,12,13,14,15].

According to de Almeida et al. (2021) [11], nearly 300 records of mosquito-associated viruses have been registered in the NT database of GenBank over the last decade. In the Americas, studies conducted in tropical and subtropical regions with a high circulation of insect-borne viruses, including Brazil, Colombia, Guadeloupe, the United States, Trinidad, Puerto Rico, have examined the viromes of various vector species [8,12,16,17,18,19,20,21]. These studies have consistently demonstrated a high diversity of viral communities associated with mosquitoes. A wide range of RNA and DNA viral families and genera have been identified, many of which appear to be regionally distributed, likely reflecting local ecological conditions and host-specific factors [2,17].

Mosquito viruses comprise a broad spectrum of virus families, such as *Togaviridae*, *Flaviviridae*, *Rhabdoviridae*, *Bunyaviridae*, and *Mesoniviridae*. These families include both arboviruses and insect-specific viruses (ISVs) [11,22,23].

In particular, the ISVs are a group of RNA and DNA viruses that naturally infect insects but cannot infect vertebrates [2,11,24]. ISVs are often found in symbiotic associations with mosquitoes in the wild and can influence many aspects of mosquito biology [25,26,27]. Several studies suggest an interaction of ISVs and related arboviruses in vectors that positively or negatively affect arbovirus infection and transmission [24,25,28,29,30,31]. For example, a *Cell fusing agent virus* (CFAV) strain, insect-specific flaviviruses (ISFVs), isolated from *Aedes aegypti* mosquitoes reduced the spread of DENV and ZIKV in *Aedes albopictus* (C6/36) cell lines. In contrast, another CFAV strain derived from an *Ae. aegypti* cell line (Aag2) has been shown to enhance DENV replication in another *Ae. aegypti* cell line (Aa20) [32,33,34,35]. Because ISVs can naturally infect and replicate in mosquitoes, and can even be transmitted from generation to generation, they can interfere with arbovirus proliferation and are harmless to vertebrates and have great potential for biocontrol [29,36]. Additionally, some ISVs are phylogenetically related to arboviruses of *Flaviviridae*, *Bunyaviridae*, *Togaviridae*, and *Rhabdoviridae* families. Consequently, it has been proposed that ISVs may represent a precursor of arboviruses [11,24,29].

The mosquito virome is closely linked to the interactions between mosquitoes, their life history, and their environment. Factors such as geographic regions, food sources (blood hosts and plant nectar), mosquito species, sex, and environmental conditions during the larval stage (diversity of microalgae or crustaceans) shape the viral composition that mosquitoes harbor [4,8,37,38] Consequently, many mosquitoes carry viruses from plants, fungi, or aquatic environments that cannot replicate in the mosquitoes themselves [1,38,39]. 

Some studies indicate that deforestation can alter mosquito abundance, sometimes increasing it, particularly for species linked to vector-borne diseases [2,3,40,41,42,43] modification can lead to a change in the microclimate, creating more favorable conditions for feeding sites or sites for immature mosquito stages. This can subsequently improve mosquito survival and reproduction [3,38,44,45]. In addition, deforestation can also lead to increased human interaction with wildlife, increasing the likelihood of human–vector contact and pathogens [3,12,42,46].The conversion of natural areas leads to biodiversity loss, which also affects the abundance and richness of parasites (each host is likely to carry its own specific pathogens) [2,11,42,47,48]. Disturbed habitats reduce the available resources from which their hosts acquire most parasites, including viruses [2,46,49].

Mexico is recognized for its high biodiversity and hosts approximately 90 mosquito species distributed across two subfamilies and 16 genera, accounting for nearly 12% of the globally described mosquito fauna [50]. The Yucatán Peninsula is an area of great biodiversity, major socio-ecological conflicts, and high levels of habitat loss and fragmentation [51]. 

This region is of particular interest, as it represents a zone of interaction between humans, wildlife, and pathogens. The emergence and re-emergence of mosquito-borne diseases such as DENV, CHIKV, and ZIKV have been documented in both humans and animals, as evidenced by molecular and serological analyses [52,53,54,55,56,57,58]. However, most of these studies have focused exclusively on known human pathogens. Over the past decade, NGS technologies have been employed to detect novel and recognized viruses specific to mosquitoes in the Yucatán Peninsula [59,60,61]. Nevertheless, the complete virome of the analyzed mosquito populations has not been described.

This study aimed to characterize the viral communities of different mosquito species collected across conserved and urban habitats in the Yucatan Peninsula. Here, we provide essential baseline data that offer insights into the viruses currently circulating in mosquitoes. This information establishes a foundation for future comparative analyses to evaluate how viral communities change over time and across habitats, as well as to explore potential interactions influencing the dynamics of arbovirus transmission.

## 2. Materials and Methods

### 2.1. Site Study

The Yucatán Peninsula is a biogeographical region in southern Mexico that includes the states of Campeche, Quintana Roo, and Yucatán. The climate is subhumid and warm tropical, with average minimum and maximum monthly temperatures between 23.6 °C and 31.6 °C and an average annual rainfall of 1200 mm. The landscape is a mosaic of habitats, including mangroves, coastal lagoons, swamps, savannas, and forests, ranging from low dry deciduous forests in the north to high moist evergreen forests in the south. The east coast is much wetter, while the north coast is much drier [58]. On the Yucatán Peninsula, between 75% and 88% of the population lives in urban areas.

Twelve sampling locations were selected across the Yucatán Peninsula—four in each state (Campeche, Yucatán, and Quintana Roo)—to represent the region’s main landscape types. Sites were categorized into four habitat types based on land cover vegetation (natural, agricultural, and urban) and human population density as follows: (a) conserved (≥60% native vegetation, minimal agricultural use, and low population density <1000 inhabitants); (b) diversified rural (∼40% native vegetation, small patches of agriculture, and low population density <1000 inhabitants); (c) rural (>40% induced vegetation with extensive monocultures); and (d) urban (>60% land covered by human settlements). Detailed habitat classifications are available in Supplementary Table S1.

Mosquito collections were conducted during June to October 2021 and March to April 2022. The map representing the habitats and locations was generated using ArcGIS Desktop 10.6 [62]. Land cover data were obtained from INEGI’s Series VII Land Use and Vegetation Map, and protected areas were overlaid using the CONANP 2023 vector layer of Natural Protected Areas.

### 2.2. Sampling Collection and Species Identification

At each site, nine BG-Sentinel traps (Biogents AG, Regensburg, Germany) were set at 300 m intervals. The traps were fitted with BG baits and 1.5 kg dry ice as a CO_2_ source. Each BG-Sentinel trap was operated for one night at each sampling location. Collected mosquitoes were identified to species, sex, and feeding status through visual inspection under a stereoscopic microscope on a chilled table based on the presence of visible blood in the abdomen. Species identification was performed using the taxonomic key of Clark-Gil and Darsie (1983), which was adapted according to the overview of the mosquito fauna occurring on the Yucatán Peninsula [50].

### 2.3. Sampling Selection and Processing

A subset of unfed female mosquitoes was selected based on the longitudinal mosquito sampling conducted in the Yucatán Peninsula, as described by García-Suárez et al. (2024) [45]. To assess the representativeness of the mosquitos analyzed, we compared its taxonomic composition with the total field captures across habitat type during the study period. An ANOSIM test based on Bray–Curtis dissimilarities yielded no significant differences (R = –0.26, *p* = 0.932), indicating that the virome analysis was conducted on a representative subset of the mosquito community. Full details of the analysis are provided in Supplementary Material S1.

The mosquitoes were grouped from the same location and capture date and stored in 1.5 mL tubes containing 300 μL RNAlater™ (ThermoFisher, MA, USA, Cat. No. AM7021) at −80 °C until further processing. The samples were processed at the Institute of Virology, Charite Medicine University in Berlin, Germany.

### 2.4. Extraction of Genetic Material

Individual mosquitoes were lysed with two 3 mm tungsten beads at 25 Hz for two minutes using the TissueLyser II (Qiagen, Corp., Valencia, CA, USA Cat. No. 85300) in 500 µL PBS (ThermoFisher, MA, USA, Cat. No. 10010023). Genetic material was extracted from 200 µL of the lysate using the MagNA Pure 96 DNA, Viral RNA Small Volume Kit (Roche, Basel, Switzerland, Cat. No. 06543588001), and a MagNA Pure 96 Instrument System (Roche; Cat. No. 06541089001). The elution volume was 50 µL according to the manufacturer’s recommendations for liquid samples or low cell content.

For genomic library construction, 10 μL from each individual extraction was combined to form pools of 1 to 5 individuals, which were then homogenized. This step aimed to reduce the high intracellular content that can inhibit sequencing reactions and contribute to genetic material degradation, ultimately decreasing the sensitivity of the analysis.

### 2.5. Genomic Libraries and Massive Sequencing

The KAPA RNA HyperPrep Kit (Kapa Biosystems, MA, USA Cat. No. KR1350-v4.21) for Illumina^®^ platforms was used to prepare genomic libraries from total RNA according to the manufacturer’s protocol. The libraries were sent to the Institute of Immunology and Genetics in Kaiserslautern, Germany, for sequencing. Sequencing data were generated using the Illumina NextSeq 1000 run at paired-end 2 × 150 bp.

### 2.6. Bioinformatic Analysis

The quality of the raw reads was initially assessed with FastQC [63]. Adapters and low-quality reads (below Q30) were removed with TrimGalore [64] to ensure high-quality paired-end reads in RRBS mode and to address biases in the final repair based on the software’s default parameters. The cleaned reads were mapped against the SILVA database [65] containing small (16S/18S, SSU) and large (23S/28S, LSU) ribosomal RNA sequences using Bowtie2 (version 2.4.4) [66] to filter out non-viral sequences.

Subsequently, the cleaned reads were assembled into contigs using the metaSPAdes genome assembler (version 3.13.1) [67] using the default settings. To identify virus-associated contigs, all assembled sequences were compared against a local viral database constructed from the NCBI nucleotide database to ensure that only viral sequences were retained [68]. Comparisons were performed using BLASTn with the following parameters: word size = 11, query coverage ≥ 80%, identity ≥ 70%, and a maximum of 20 hits per query. A hit was considered significant if it had an E-value smaller than 1 × 10⁻⁵.

For taxonomic level, viral operational taxonomic units (vOTUs) were defined based on contigs with (i) a minimum length of 100 base pairs, (ii) a minimum bit-score of 50, (iii) an alignment identity threshold of ≥60% for viral family-level assignments and ≥80% for species-level assignments, (iv) an E-value threshold of 1 × 10⁻⁵, (v) a top-percent filter within 10% of the best hit, and (vi) a minimum support of three reads per contig. Taxonomic classification was performed using the Lowest Common Ancestor (LCA) algorithm in MEGAN (v6.24.20), and only contigs classified under viral taxa were retained [69]. 

The resulting vOTUs were used to characterize viral community composition and diversity across mosquito species and habitats. MEGAN was also used to perform phylogenetic tree and hierarchical clustering analysis based on species-level taxonomic profiles, allowing for comparison of viral community composition across samples.

### 2.7. Virus Abundance and Diversity Metrics

Relative viral abundances based on vOTUs were then compared across samples using the normalized read counts and visualized with the ggplot2 package (v3.4.3) in R [70]. Alpha viral diversity was assessed based on the number of viral families (viral family richness) and their relative abundance in mosquito samples from different habitats; the Shannon diversity index was used to consider both richness and abundance, which was calculated using the vegan library (version 2.6-4) in R [71]. Statistical differences in alpha diversity between habitats were evaluated using Kruskal–Wallis test in R Studio with the ggpubr package (version 0.6.0) [72].

Beta diversity, representing differences in viral communities between habitats, was estimated using the Bray–Curtis dissimilarity index. The analysis was performed with the betapart package (v1.6) in R [73]. Principal Coordinate Analysis (PCoA) was performed based on the Bray–Curtis dissimilarity matrix using the vegan package (v2.6-4) in R [71]. Permutational analysis of variance (PERMANOVA) was performed using adonis function (vegan v2.6-4) [74] to identify significant differences in viral community between habitats and mosquito species. For pairwise comparisons between groups, we applied multiple testing correction using the Bonferroni method at 999 permutations.

Finally, we performed an analysis of the differential abundance of virus families of mosquito species and habitat types. We employed linear discriminant analysis (LDA) in conjunction with effect size (LEfSe), with an LDA score threshold of 2 and *p* < 0.05 for significance. For this analysis, the ldamarker function from the microbial package (v0.0.22) in R [75] was used, which includes Kruskal–Wallis tests and LDA to identify significant associations.

## 3. Results

**Figure 1:**
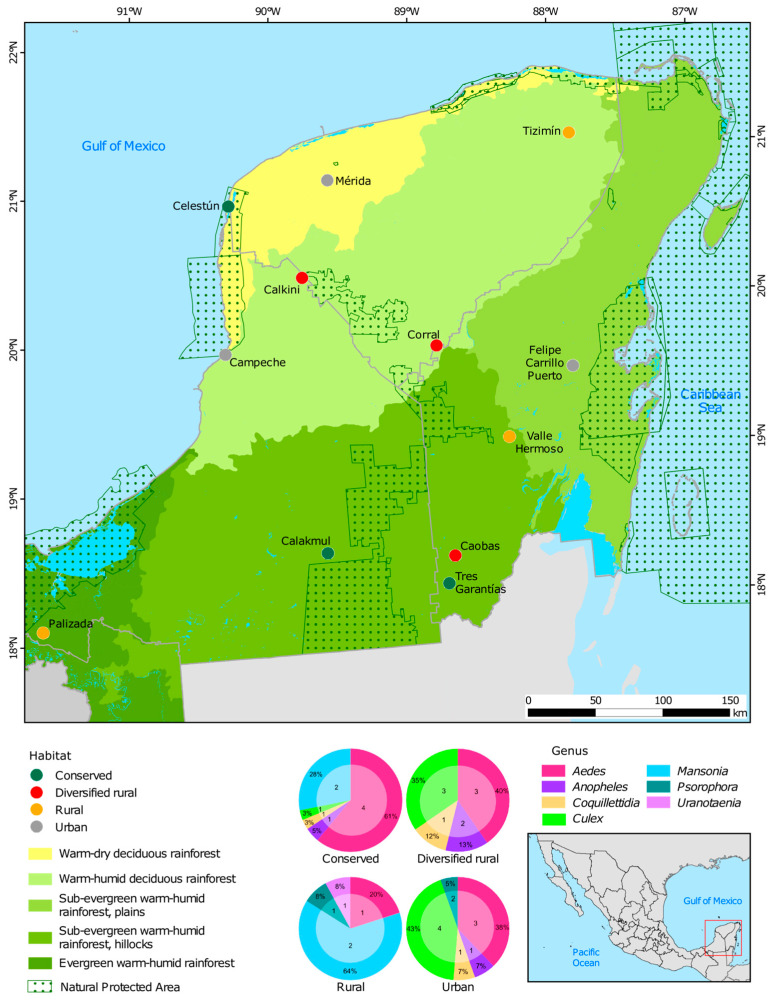
Location of the 12 sampling sites, classified into four habitat types based on cover vegetation and human population density in the Yucatán Peninsula: conserved (green), rural (ochre), diversified rural (red), and urban (gray). For each habitat, the corresponding percentage of mosquito genera is shown, along with the number of analyzed species within each genus.

A total of 215 mosquitoes from 20 species across seven genera (*Aedes, Culex, Anopheles, Psorophora, Mansonia, Uranotaenia,* and *Coquillettidia)* were analyzed. Aedes (43%) and Culex (24%) were the most abundant. Figure 1 shows the percentage of samples by genus and the total number of species analyzed by habitat.

Samples were grouped into 61 pools of 1–5 mosquitoes by species and habitat. A total of 509.6 million sequence raw reads were obtained, yielding an average of 8.35 million reads per library. Detailed information of the samples, collection sites, habitat classification, and individual sequence accession numbers is available in Supplementary Table S1.

### 3.1. Diversity of the Viral Community

**Figure 2:**
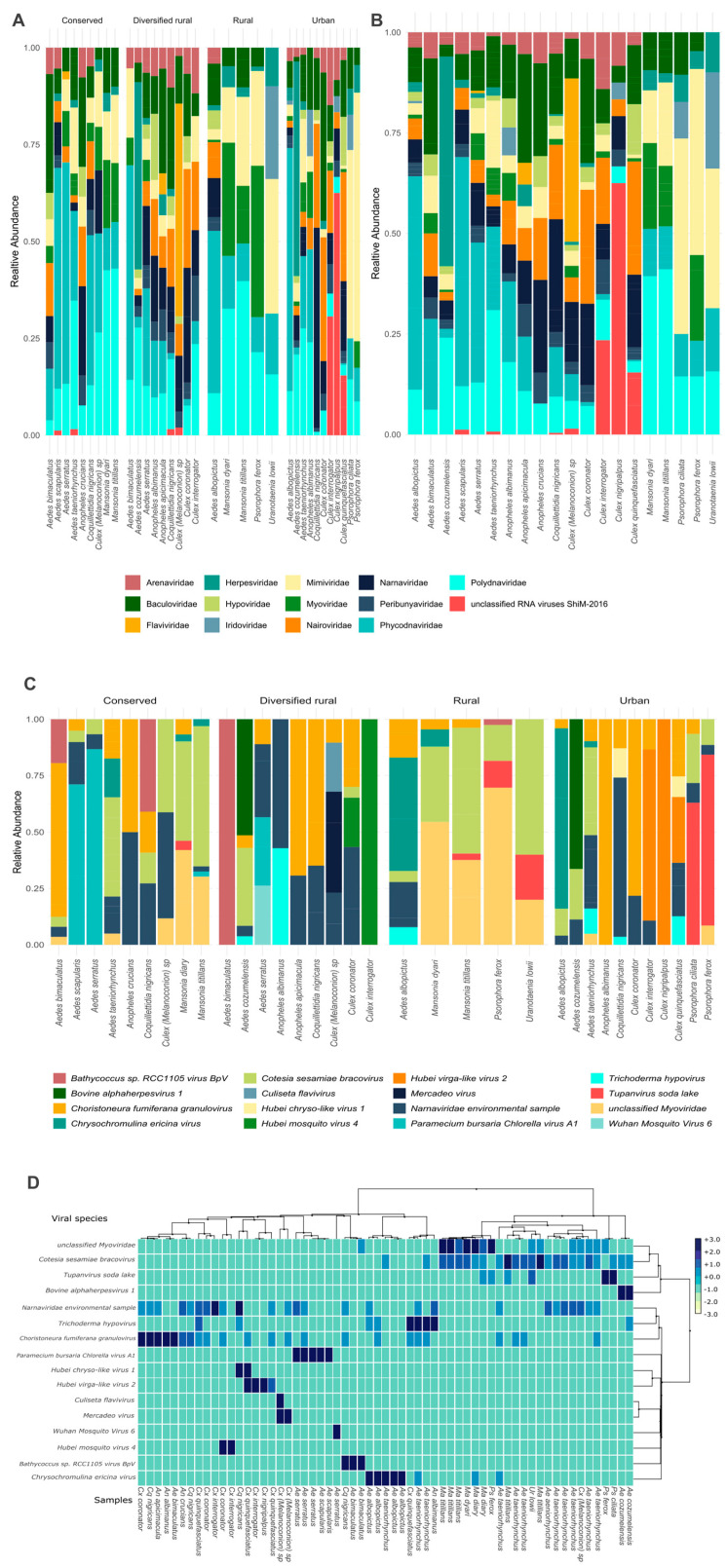
Relative abundance based on vOTUs at family and species level identified in mosquito species. (**A**) In relation to the mosquito species in the four habitats. (**B**) In relation to the mosquito species analyzed, regardless of the habitat. It is interesting to note the graphical differences between the species in the different habitats as well as the differences between the mosquito species. (**C**) Relative abundance of viral species identified in mosquito samples by habitat. (**D**). Hierarchical clustering and heatmap of viral species composition across mosquito samples. The dendrogram illustrates the grouping of samples based on taxonomic profiles at the species level.

The vOTUs identified in mosquitoes from the four habitats comprised RNA and DNA virus families, including viruses associated with a diverse range of hosts. A total of 16 viral species were identified, belonging to 13 viral families classified and one group determined like unclassified RNA viruses ShiM-2016 previously identified in insect species [76]. BLASTn results are included in Supplementary Table S2. The most abundant belonged to the family *Phycodnaviridae* (*Paramecium bursaria chlorella virus 1*, *Bathycoccus sp. RCC1105 virus BpV1*) and *Mimiviridae* (*Tupanvirus soda lake*, *Chrysochromulina ericina virus*), which are both primarily associated with the infection of algae and protists. Additionally, viral contigs classified into families known to infect insects and plants were detected, including *Polydnaviridae* (*Cotesia sesamiae bracovirus*), *Iridoviridae*, *Baculoviridae* (*Choristoneura fumiferana granulovirus*), and two viral species; *Hubei mosquito virus 4* and *Hubei virga-like virus 2* belong to unclassified RNA viruses ShiM-2016. Furthermore, viral families associated with bacterial hosts, such as *Myoviridae*, and fungal hosts, including *Hypoviridae* (*Trichoderma hypovirus*), were also present. Similarly, we detected *Flaviviridae*, *Nairoviridae*, and *Peribunyaviridae* families, which include species recognized as arboviruses and were identified in *Aedes* and *Anopheles* mosquitoes collected from urban and diversified rural habitats. Likewise, specific insect-associated flaviviruses, including *Culiseta flavivirus* and *Mercadeo virus*, were identified in mosquito *Culex (Melanoconion) sp*. from diversified rural habitats. Figure 2A,C represent the relative abundance of viral families and viral species, respectively, identified in mosquito samples across the four habitats.

Additionally, the relative abundance of viral families, grouped by mosquito genus and habitat, is presented in Supplementary Figure S1. *Culex* mosquitoes from urban areas exhibited a high relative abundance (24.6%) of sequences related to ShiM-2016 *unclassified RNA virus*, including *Hubei virga-like virus 2*, which were detected in *Culex interrogator* and *Culex nigripalpus*. In contrast, *Hubei mosquito virus 4* identified in *Culex interrogator* and *Culex coronator* were much less abundant in diversified rural areas (0.7%). *Herpesviridae* showed higher relative abundances in *Aedes* mosquitoes from diversified rural (10.8%) and urban (8.1%) areas compared to very low abundances in conserved (1.3%) and rural (1.2%) areas. The analysis at the viral species level identified the presence of *Bovine alphaherpesvirus 1* in *Aedes cozumelenzis* from both diversified rural and urban habitats.

The relative viral abundance based on the vOTUs of each mosquito species was grouped and analyzed, regardless of the habitat classification to assess differences. Figure 2B provides a comparative overview of the virome across the twenty species, showing that *Mansonia titillans* and *Mansonia dyari* exhibited similar viral family composition and abundance. A similar pattern was observed for *Anopheles apicimacula* and *Anopheles crucians*.

Hierarchical clustering identified two main viral groups among mosquito samples. As shown in Figure 2D, *Culex* and *Aedes* mosquitoes mainly harbored insect-associated viruses clustered closely (left side), indicating similar viral community compositions. In contrast, samples from *Mansonia* and *Psorophora* formed distinct clusters in the dendrogram, reflecting differences in their virome composition, particularly in relation to plant- and algae-associated viruses. The viral community identified in mosquito species across different habitats illustrates their wide distribution in diverse ecosystems. The identified viruses include both insect-associated viruses and those capable of infecting vertebrates. Supplementary Table S3 provides a detailed overview of the identified virus families and viral species, highlighting their ecological and epidemiological significance. Additionally, a phylogenetic tree illustrating the relationships among the viral species, along with the hosts in which they have been reported, is presented in Supplementary Figure S2.

**Figure 3:**
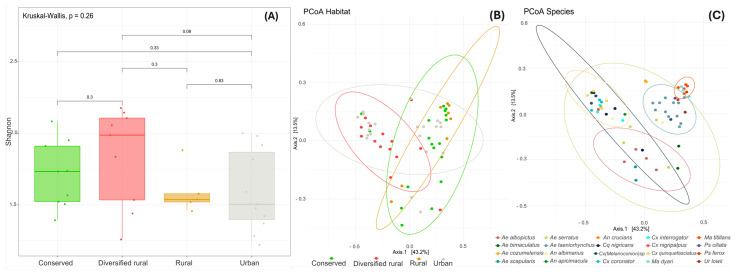
Diversity analysis of viral communities across habitats. (**A**) The Shannon diversity index indicates that diversified rural areas exhibit the highest viral diversity. No significant differences in alpha diversity of the habitats. (**B**) Principal Coordinate Analysis (PCoA) showing clustering of viral communities across four habitat types. (**C**) PCoA clustering of viral communities grouped by mosquito species. Axis 1 explains 43.2% of the variability, and Axis 2 explains 13.5%.

The alpha diversity of the viromes of mosquito populations circulating in all four habitats showed a discrepancy in the diversity. The highest diversity was observed in mosquitoes of the diversified rural habitat, followed by the conserved habitat, while the urban and rural habitats exhibited lower values. However, these differences were not statistically significant (*p* = 0.26), Figure 3A. The mosquito species with the highest viral diversity in the diversified rural area were *Aedes serratus* and *Anopheles apicimacula*. 

The beta diversity value (0.893) revealed a high degree of variability in viral community composition among mosquito species across different habitats. Additionality Principal Coordinate Analysis (PCoA) based on Bray–Curtis dissimilarity showed overlapping viral community compositions among the four habitat types, suggesting similarities in viral diversity across environments (Figure 3B). This overlap may indicate shared environmental conditions or ecological factors that influence the viral community structure within these habitats. Meanwhile, as shown in the Figure 3C, mosquitoes of the same species formed distinct clusters, particularly in *Aedes taeniorhynchus*, *Mansonia titillans*, and *Mansonia dyari*, which revealed characteristic viral community compositions for these species. Notably, *Aedes serratus* exhibited the most variable viral composition, reinforcing earlier findings that this species harbors the highest viral diversity.

The results of the influence of mosquito species and habitat on viral community composition indicate that mosquito species accounted for 66.5% of the variation (*p* = 0.001), while habitat explained only 3.2% of the variation (*p* = 0.082). The interaction between mosquito species and habitat contributed 12.7% of the variation (*p* = 0.007), suggesting that mosquito species and the interaction in the habitat play significant roles in shaping viral community composition. However, pairwise comparisons did not reveal statistically significant differences between specific groups.

### 3.2. Differential Abundance of Viral Families

**Figure 4:**
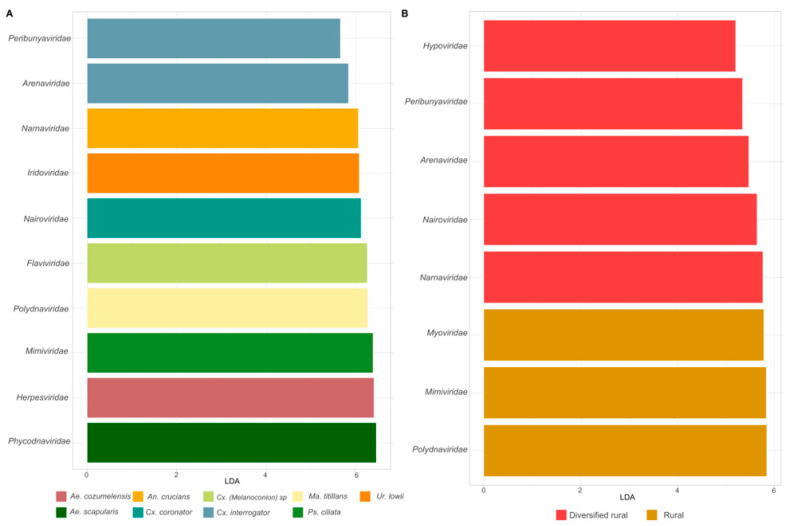
Differential abundance of virus families in different mosquito species and habitats. (**A**) Viral families with different frequencies among mosquito species, including *Flaviviridae* and *Mimiviridae.* (**B**) Viral families with different abundances among habitats, with *Polydnaviridae* and *Hypoviridae* enriched in rural and diversified areas. Families such as *Arenaviridae* and *Nairoviridae* overlap in both categories.

To identify the viral taxa driving the observed variability, we analyzed the differential abundance of viral families across mosquito species and habitats. Figure 4A,B present the LDA scores corresponding to differential abundance, highlighting several viral families with significantly different abundances among mosquito species and habitats. Taxa associated with insects included *Flaviviridae* in *Culex (melanoconion*) sp., *Polydnaviridae* in *Mansonia titillans*, and *Iridoviridae* in *Uranotaenia lowii*. Additionally, viral families associated with algae and protozoan hosts such as *Mimiviridae* and *Phycodnaviridae* were identified in *Psorophora ciliata* and *Aedes scapularis*, respectively.

Notably, the genus *Culex* exhibited the highest number of differentially abundant taxa. Within this genus, *Culex interrogator* was significantly associated with the families *Arenaviridae* (LDAscore 5.8) and *Peribunyaviridae* (LDAscore 5.66), while *Culex coronator* showed a significant association with *Nairoviridae* (LDAscore 6.12). Viral families such as *Polydnaviridae*, *Mimiviridae*, and *Myoviridae* were more abundant in mosquitoes from rural habitats, while *Narnaviridae* and *Hypoviridae* were more commonly associated with mosquitoes from diversified rural habitats.

This information contributes to our understanding of the role of specific viral families in shaping the virome of mosquito species. Complete LDA scores for the differential abundance analysis of each mosquito species and habitat are provided in Supplementary Table S4.

## 4. Discussion

In this study, we describe the viral community of twenty mosquito species from twelve localities classified in four different habitats on the Yucatán Peninsula, Mexico. We identified sixteen viral species associated with several hosts. Although no sequences corresponding to recognized arboviral species were identified, sequences belonging to viral families that include arboviruses were detected. In addition, contigs were identified as insect-specific viruses, as well as viruses associated with other organisms, such as plants and algae.

All viral contigs identified in this study have been previously detected in mosquito viromes, confirming the consistency of our results with prior research. This underscores the utility of metagenomic approaches for characterizing the virome associated with mosquitoes [13,38,40,77,78]. 

Thanks to this technique and ecological analyses, it has been possible to determine that the invertebrate microbiome is influenced by various factors, such as larval habitat, environmental conditions, and host species, as well as viruses that may originate from food sources or parasitic infections [20,39]. In our study, the predominance of *Phycodnaviridae*, *Polydnaviridae*, *Baculoviridae*, and *Mimiviridae* across mosquito species and habitats emphasizes their ecological importance. The presence of these viral families in mosquitoes is likely influenced by both host traits and environmental factors. For instance, the detection of viruses associated with plant infections suggests the existence of a potential ecological relationship between viruses infecting plants and mosquitoes that feed on them [8].

Additionally, aquatic habitats are essential for mosquito larval development and may play a significant role in shaping the virome composition [79,80]. We observed a high abundance of *Choristoneura fumiferana granulovirus* from *Baculoviridae*, which is known to infect a limited number of host insects, mainly from the orders Lepidoptera and Hymenoptera, with larvae being particularly susceptible to infection. Due to its ability to target pest species, this virus is considered a promising biological insecticide; however, further studies are needed to better understand its role in mosquitoes [81,82]. Interestingly, this virus has previously been reported abundantly in viromes of *Aedes albopictus* populations from India [83]. In our study, *Choristoneura fumiferana granulovirus* was predominantly detected in *Aedes albopictus*, *Culex coronator*, *Coquilletidia nigricans*, and *Anopheles nigricans*, suggesting ecological interactions influencing its distribution.

The presence of viruses capable of infecting protists and algae in adult mosquitoes indicates that these organisms may be replicating actively in the host or may be continuously exposed to environmental sources throughout the life cycle of the mosquitoes [26]. However, based on our results, it is not possible to conclude that the presence of sequences from these and other viruses indicates active replication within mosquitoes. Additional analyses are required to confirm viral replication, such as the detection of viral mRNA or in vitro replication assays. These approaches would help to explore potential correlations with viruses shared between mosquitoes and other hosts [84]. 

We identified viral contigs associated with *Herpesviridae* and members of the *Bunyavirales* order, such as *Arenaviridae*, *Nairoviridae*, and *Peribunyaviridae*. The arboviruses within these families are primarily transmitted by *Aedes* and *Culex* mosquitoes, highlighting their critical role as primary vectors. In our study, the criteria used to identify viral species, particularly fragment size and percent identity, were not enough to confidently assign sequences to specific species within these families. This limitation, coupled with the lack of comprehensive reference data for many other viral taxa, led us to conduct the study at this level. It underscores the importance of advanced NGS methodologies, such as targeted enrichment protocols tailored to the identified viral families [85]. To address these constraints and ensure reliable taxonomic classification, our analyses were performed at the family level.

The detection of bovine alphaherpesvirus type 1 in our results is likely linked to the feeding behavior of mosquitoes and their proximity to human settlements where these hosts are present, making them part of the available blood meal sources. Previous studies have documented that mosquitoes frequently feed on bovine blood. For instance, Mwanga et al. (2024) [86] reported that 45.2% of *Anopheles* mosquitoes had consumed bovine blood compared to 9% that fed on human blood and 3.7% on other sources. Previous studies have identified several species of herpesviruses in high abundance in mosquitoes, including those associated with humans, suggesting that herpesviruses infect a wide range of animals and can potentially be transmitted to insects [8,87,88]. Emphasizing the complex ecological interactions between mosquitoes and vertebrates highlights the potential role of mosquitoes as an accidental host of herpesviruses [89]. 

*Flaviviridae* includes both arboviruses and insect-specific viruses, and their presence in natural habitats highlights the remarkable diversity of the mosquito virome and its ecological significance [43,78,90]. Our analysis revealed the presence of *Mercadeo virus*, an insect specific flavivirus associated with *Culex Flavivirus* (CxFV) reported in *Culex* mosquitoes. To our knowledge, this is the first report of *Mercadeo* virus in *Culex (Melanoconion) sp*. in Mexico (GenBank access PV059844, PV059845). Carrera et al. (2015) [91] described the phylogenetic relationship between *Mercadeo virus* and CFAV, which are both considered CxFVs. Infection with CxFV can either enhance or block the ability of mosquitoes to be infected by other pathogenic flaviviruses. Additionally, CxFV-positive mosquitoes exhibit differences in flight activity, which may reduce their contact with arbovirus amplification hosts. These findings raise important questions about the evolutionary origins of insect-specific viruses (ISVs) [24,91,92]. 

The diversity of mosquito viromes was different across the habitats. For example, diversified rural habitats exhibited the highest diversity of viral families, while rural and urban habitats showed the lowest. However, these differences were not statistically significant, indicating that viral diversity cannot be directly linked to the diversity of the vector community. This could be due to the heterogeneity in the number and identity of mosquito species analyzed, which may not fully represent the entire vector community. The transformation of natural habitats into urban environments has been shown to alter community structure, population density, and mosquito species diversity, leading to ecological disturbances and biodiversity loss, including microbiome diversity [2,38,41].

According to our results, variations in the viral community at the family level were explained by mosquito species. Even species within the same genus maintained a viral community. Most insect-specific viruses (ISVs) and persistent viruses are thought to constitute the insect core virome, which exhibits relative stability among individuals of the same species [93]. However, Coninck and Matthijnssens (2024) [94] propose that the concept of a mosquito virome must be approached carefully, as it is influenced by other factors, including the biology of mosquito species and geographic region, source food, gut microbiota, and environmental variations, and may naturally fluctuate with time, climate, and other ecological factors [4,27,95,96]. Consequently, extrapolating our findings to other mosquito populations worldwide may not always be appropriate, given the geographical and ecological factors that shape viral diversity [76,97]. 

The advent of NGS technology has facilitated the detection of not only arboviruses in mosquitoes but also other insect associated viruses and viruses related to other hosts. Consequently, this has resulted in the identification of numerous novel viruses, the ecology of which remains to be fully elucidated [15]. LEfSe analysis revealed significant differences in the abundance of viral families in mosquito species, indicating that specific viral families are more prevalent in particular hosts. Notably, the most abundant viral taxa belonged to families commonly associated with invertebrate and plant hosts, including *Flaviviridae*, *Polydnaviridae*, and *Iridoviridae*. This finding could indicate that mosquito viromes are specific according to the species and habitats. The functional roles and interactions of these viruses might be studied, together with ecological associated factors, such as host characteristics that play a significant role in shaping the distribution of these viral families [93,98].

## 5. Conclusions

This study provides the first virome characterization of mosquito species from diverse habitats in the Yucatán Peninsula, Mexico, using next-generation sequencing. Our findings highlight the significant role of mosquito species identity in shaping viral composition, with closely related species exhibiting similar viromes. These results underscore the influence of mosquito-specific traits on viral diversity. Future research integrating vector community structure is essential to elucidate the ecological dynamics of mosquito–virus interactions. This work establishes a baseline for the surveillance of mosquitoes and associated viruses.

## Data Availability

Genetic data: Raw sequence reads are deposited in the SRA (BioProject PRJNA1157373), Mercadeo virus GenBank access PV059844, PV059845. Sample metadata: Related metadata can be found in Supplementary Table S1.

## Supplementary Materials

The following supporting information can be downloaded at https://www.mdpi.com/article/10.3390/v17060758/s1. Figure S1: Heatmap illustrates the relative abundance of viral families observed of mosquito genera across habitat types. Some mosquito genera in specific habitats exhibit high or low abundances of certain viral families; Figure S2: Phylogenetic tree show relation of viral species contigs recovered; Table S1: Mosquito samples and habitat classification; Table S2: BLASTn results; Table S3: Viral families and species identified in mosquitos samples; Table S4: LDA scores for the differential abundance analysis of each mosquito species and habitat.

## Abbreviations

The following abbreviations are used in this manuscript:

NGS	Next-generation sequencing
DENV	Dengue virus
CHIKV	Chikungunya virus
WNV	West Nile virus
ZIKV	Zika virus
ISVs	Insect-specific viruses
CFAV	*Cell fusing agent virus*
ISFVs	Insect-specific flaviviruses
vOTU	Viral operational taxonomic unit
LCA	Lowest Common Ancestor
PCoA	Principal Coordinate Analysis
PERMANOVA	Permutational analysis of variance
LDA	Linear discriminant analysis
LEfSe	Effect size
CxFV	Culex Flavivirus
