# Supplementary material for: Characterization of the Virome in Mosquitoes Across Distinct Habitats in the Yucatán Peninsula, Mexico
Source: Viruses. 2025 May 26;17(6):758. doi: 10.3390/v17060758 (PMC12197341; doi:10.3390/v17060758)
Supplement: Supplementary file 1 [file viruses-17-00758-s001.zip › Caption supplementary files.pdf]

Caption supplementary files

Supplementary Figure S1. Heatmap illustrates the relative abundance of viral families observed of mosquito genera across habitat types. Some mosquito genera in specific habitats exhibit high or low abundances of certain viral families.

Supplementary Figure S2. Phylogenetic tree show relation of viral species contigs recovered.

Supplementary table S1. Mosquito samples and habitat classification.

Supplementary table S2. BLASTn results

Supplementary table S3. Viral families and species identified in mosquitos samples

Supplementary Table S4. LDA scores for the differential abundance analysis of each mosquito species and habitat
