# Supplementary figures and images for: Characterization of the Virome in Mosquitoes Across Distinct Habitats in the Yucatán Peninsula, Mexico
Source: Viruses. 2025 May 26;17(6):758. doi: 10.3390/v17060758 (PMC12197341; doi:10.3390/v17060758)

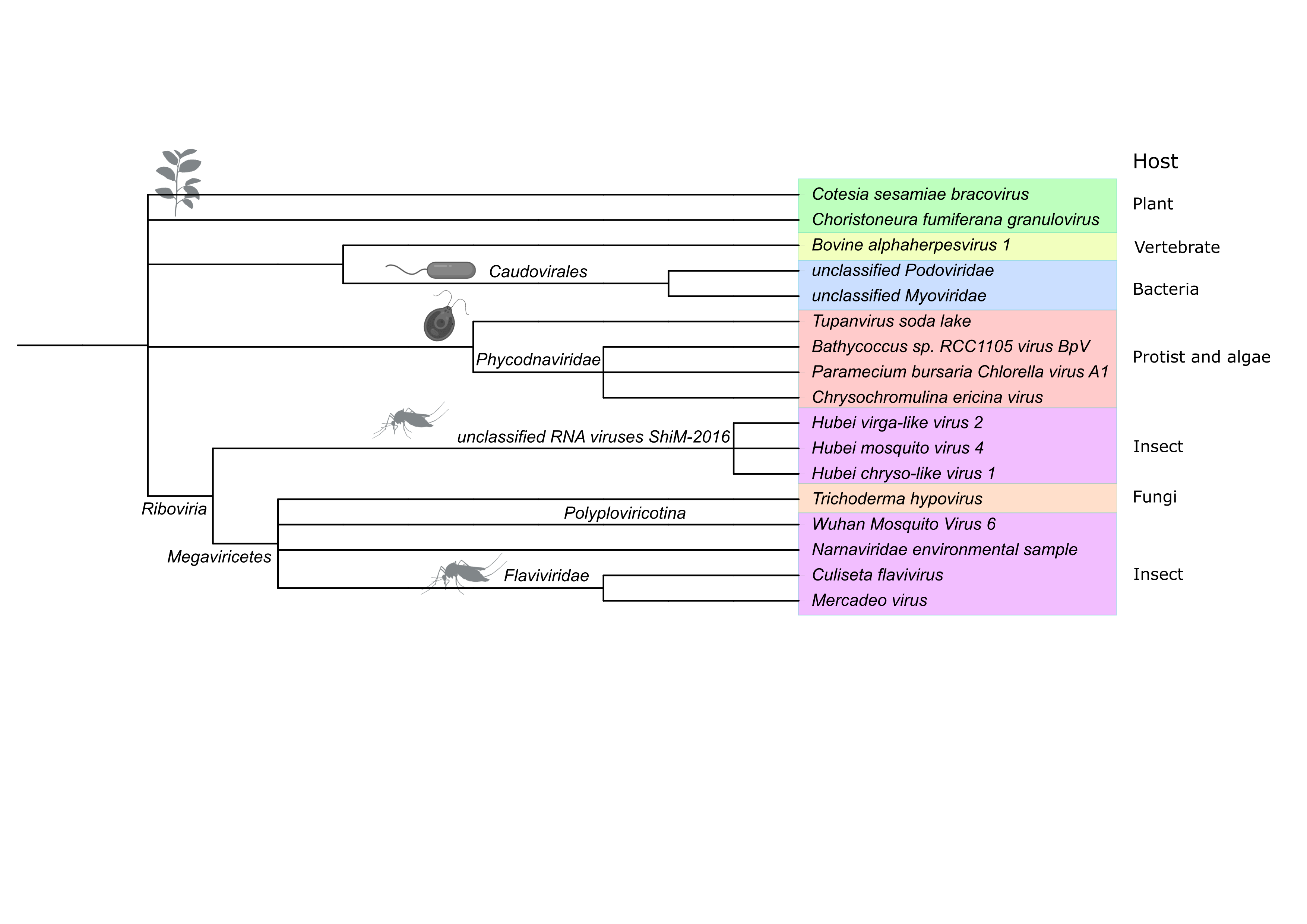

Supplement: Supplementary file 1 [file viruses-17-00758-s001.zip › Supplementary figure S2.png]
